# Handgrip Strength to Predict the Risk of All-Cause and Premature Mortality in Korean Adults: A 10-Year Cohort Study

**DOI:** 10.3390/ijerph19010039

**Published:** 2021-12-21

**Authors:** Junghoon Kim

**Affiliations:** Sports and Exercise Medicine Laboratory, Korea Maritime and Ocean University, 727 Taejong-ro, Yeongdo-Gu, Busan 49112, Korea; junghoonkim@kmou.ac.kr; Tel.: +82-51-410-4792

**Keywords:** handgrip strength, muscle strength, mortality, premature mortality, sarcopenia

## Abstract

The prospective association of muscular weakness with the risk of all-cause and premature mortality in a general population remains unknown. The aim of this study was to investigate the prospective effects of handgrip strength and muscular weakness on risk for all-cause and premature mortality over 10 years using a large nationwide sample of Korean adults. The study participants included 9229 middle and older adults (4131 males and 5098 females), using data from the Korean Longitudinal Study of Ageing 2006–2016. Muscular strength was measured using handgrip strength. Muscle weakness was defined using the sex-specific handgrip strength index based on the Asian Working Group on Sarcopenia in Older People (AWGSOP). The primary outcome was all-cause and premature mortality assessed based on the death certificate. The hazard ratio (HR) for all-cause mortality was negatively associated with level of handgrip strength independent of potential confounding factors (HR: 2.06, 95% confidence interval [CI]: 1.62–2.63 for lowest quartile vs. highest quartile). When examined using muscle weakness defined using the AWGSOP diagnosis, the mortality was 1.56 times higher in the weak group (HR: 1.56, 95% CI: 1.36–1.78). We also found that risk of premature mortality was observed in the lowest quartile (HR: 2.34, 95% CI: 1.80–3.05) and the muscle weakness group (HR: 1.80, 95% CI: 1.52–2.13) in the fully adjusted model. Our 10-year prospective cohort study showed that handgrip strength and muscle weakness are strongly associated with an increased risk of all-cause and premature mortality in healthy middle-aged and older adults.

## 1. Introduction

Biological aging is characterized by changes in body composition and physical function. Sarcopenia, the loss of muscle mass or strength as a result of aging, has been linked to an increased risk of chronic disease, physical impairment, cognitive dysfunction, hospitalization, and mortality [1,2,3,4,5,6]. Therefore, screening this population is an important intervention technique that promotes public health.

Muscular strength, as assessed by handgrip strength, is a common way to test simple muscle function and is an excellent indication of decline in physical function and biological aging [7]. There is mounting evidence that low muscle strength makes it easier to assess functional and clinical health outcomes [3,5,8]. Handgrip strength, frequently used to evaluate muscle strength, reflects the strength of the whole body. It has the advantage of being simple and safe to assess in older individuals [7]. Reduced handgrip strength has been linked to adversity in elderly persons in previous research. Previous studies in older people have shown that reduced handgrip strength can predict adverse health events such as cognitive decline, disability, frailty, falls, hospitalization costs, and mortality [1,9,10,11], and it is commonly used as an objective measurement of muscular strength in epidemiological studies [7]. Low handgrip strength was also utilized as a surgical definition of sarcopenia by the Asian Working Group on Sarcopenia in Older People (AWGSOP) so that it may be a simple test to examine older people without evaluating muscle mass [12].

Premature mortality is defined as death that occurs before one’s expected life expectancy. Premature mortality from noncommunicable diseases (NCDs) results in reduce productivity and have an economic impact [13]. When compared to other diseases, premature death is the leading cause of death among the youngest and most productive age group and a leading cause of potential loss of lifespan [13,14]. In the general population and in those with type 1 diabetes, higher levels of physical activity or cardiorespiratory fitness are linked to a lower risk of premature death [15,16,17]. Lower level of handgrip strength has been found to be a predictor of death in the elderly in several investigations [1,18,19]. Furthermore, meta-analysis studies have revealed that mortality in sarcopenia is higher than in non-sarcopenia [20]. Previous research, on the other hand, was conducted on elderly persons or a local population. It remains unclear whether muscle weakness contributes to the early mortality rate in the general population including middle-aged people [13]. Therefore, in the present study we investigated the prospective association between level of handgrip strength and the risk of all-cause and premature mortality in a general population of Korean middle-aged and older adults using data from a national cohort study over 10 years.

## 2. Materials and Methods

### 2.1. Participants

For the present study, we used data from the Korean Longitudinal Study of Ageing (KLoSA). The KLoSA is an ongoing cohort study of a representative sample of Korean adults aged 45 years or older [21]. The goal of the KLoSA is to collect basic data that may be utilized to inform and build social and economic policies in an ageing society [22]. In 2006, a total of 10,254 people took part in the baseline survey, which was performed using the Computer Assisted Personal Interviewing method, and is being repeated every two years. Individuals with severe injury or pain were excluded (N = 818) while determining whether it was possible to measure the handgrip strength. In this study, we included participants who completed handgrip strength tests (N = 9436) to evaluate the risk of all-cause mortality. We also excluded 206 participants who had missing data for sociodemographic and lifestyle related variables used as covariates. Therefore, a total of 9229 participants (4131 male and 5098 female) were included.

### 2.2. Measurement of Handgrip Strength

We used the handgrip strength index as a maximum skeletal muscle strength to describe reduced muscular strength and weakness. Each participant’s handgrip strength was measured twice using a dynamometer (Model No: 6103, TANITA, Tokyo, Japan) in a standing posture with their elbow at their side and flexed at right angles, as well as a neutral wrist position. We estimated the muscular strength index as the average of both hands’ maximum handgrip strength [23,24]. Relative handgrip strength (%) was calculated by correcting body weight by handgrip strength multiplied by 100. Muscle weakness was defined as handgrip strength of <26 kg for males and <18 kg for females, based on the diagnosis of the Asian Working Group for Sarcopenia [12]. To investigate the association of different levels of handgrip strength on all-cause and premature mortality, we categorized participants into quartiles of handgrip strength by sex (<28.5, 28.5–32.7, 32.8–37.2, and ≥37.3 kg for male, <17.0, 17.0–19.9, 20.0–22.9, and ≥23.0 kg for female).

### 2.3. Clinical Health Conditions

Clinical health status was assessed in this study, including obesity, hypertension, diabetes, cardiovascular disease (CVD), stroke, and cancer. The body mass index (BMI) was employed to define obesity (BMI). Based on an Asian reference, BMI was computed from body weight and height (weight/height^2^) and classified into two groups: normal (BMI < 25 kg/m^2^) and obese (BMI ≥ 25 kg/m^2^) [25]. Hypertension, diabetes, CVD, and stroke were diagnosed based on self-reported medication use or a history of physician diagnosis.

### 2.4. Mortality

The major endpoint in this study was the risk of all-cause mortality, which was measured over the follow-up period from 2006 to 2016. Family interviews gave the information for the death certificate. The date and cause of death were reviewed and coded using the death certificate. The period from baseline to death was calculated in months. We also defined a premature death as someone who died sooner than the average life expectancy in the Korean population (79.3 years for men, 85.4 years for women) in 2016 [26].

### 2.5. Assessment of Other Variables

In this study, we considered demographics (age, gender, education level, and household income), health-related behaviors (physical activity, smoking status, and alcohol consumption), and clinical health conditions as potential confounding factors (hypertension, diabetes, and history of CVD and stroke). The education level was divided into three categories: middle school, high school graduation, and some college. Physical activity was measured using a questionnaire that asked participants about the frequency (days/week) and duration of their physical activities (min).

We also computed total physical activity time based on frequency and duration in minutes per week. Participants were divided into two groups based on the current guidelines for Korean adults: <150 or ≥150 min per week [27]. None, light drinker, moderate drinker, and heavy drinker were the four categories of alcohol consumption. Smoking status was divided into three categories: never, former smoker, and current smoker.

### 2.6. Statistical Analysis

For data analysis, we used the statistical analysis program R ver. 4.1.2 (R Foundation for Statistical Computing, Vienna, Austria) [28]. All statistical significance was considered as a *p*-value of less than 0.05. Baseline participant characteristics were presented as mean ± standard error (SE) or number (%, percentages). To compare baseline characteristics according to level of handgrip strength, we used the analysis of variance (ANOVA) for continuous variables with Bonferroni’s post-hoc analysis and chi-squared (*X*^2^) tests for categorical variables. We used Cox-proportional hazard models to predict the risk of all-cause and premature mortality. We also calculated the hazard ratio (HR) and 95% confidence interval (CI) according to handgrip strength levels and muscle weakness. We adjusted for age, sex, education level, smoking status, alcohol consumption, MVPA, and clinical health conditions such as obesity, hypertension, diabetes, and the history of CVD stroke and cancer.

## 3. Results

The participants’ characteristics at baseline are shown in Table 1. In this study, the mean period of follow-up was 9.38 years. A total of 1269 participants died during the 10 years follow-up period. The mean age was 60.71 years at baseline. Overall, 4131 (44.8%) participants were males, 5098 (55.2%) participants were females, 1833 (19.9%) were current smokers, and 556 (6.0%) were heavy alcohol drinkers. Mean handgrip strength was 32.52 (±0.11) kg for male and 19.86 (±0.07) kg for female. Moreover, relative handgrip strength corrected by body weight was 49.63 (±0.16)% for male and 35.16 (±0.13)% for female. In total, 2675 (29.0%) participants performed ≥150 min/week of physical activity as recommended by current guidelines. In addition, 2235 (24.22) of the total, 651 (15.76%) males and 1584 (31.07) females, had weakness of muscular strength (Table 1).

Table 2 shows the baseline characteristics of participants according to category of handgrip strength levels. Age and BMI differed significantly between categories of handgrip strength levels (all *p* < 0.001). The percentage of male population, education level, smoking status, alcohol consumption, and MVPA were different across the category of handgrip strength levels (Table 2). We also found significant differences in the medical health condition, including obesity, hypertension, diabetes, CVD, stroke, and cancer (Table 2).

Compared with the not weak group, a lower survival probability was observed in weakness of muscular strength. Table 3 presents the results for the association between the time to mortality and the level of handgrip strength and muscle weakness using the Cox proportional hazards model. The mortality rate per 1000 person-years was decreased according to handgrip strength levels (Q1: 33.1, Q2: 14.1, Q3: 6.9, and Q4: 4.0 per 1000 person years). The risk of mortality was significantly higher in the lowest quartile compared with the highest quartile (reference) after adjusted for full covariates (HR: 2.06, 95% CI: 1.62–2.63, Table 3). Similarly, a lower level of relative handgrip strength corrected by body weight was associated with higher HR for morality. In addition, we also investigated the association of muscle weakness diagnosed using AWGSOP sarcopenia diagnosis and mortality. We found a higher risk of mortality in muscle weakness compared with not weak group (HR: 1.56, 95% CI: 1.36–1.78).

In this study, we also investigated the effect of level of handgrip strength and muscle weakness on the higher risk of premature mortality (Table 4). The elevated risk of premature mortality was observed in the lowest quartile (HR: 2.34, 95% CI: 1.80–3.05) and the weak group (HR: 1.80, 95% CI: 1.52–2.13) in the fully adjusted model (Table 4).

**Figure 1:**
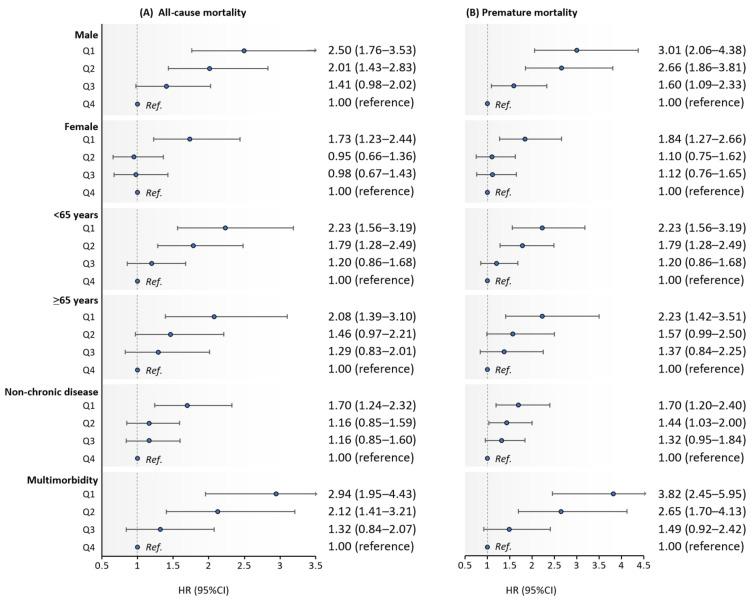
Results for the associations of the handgrip strength and weakness group on time to all-cause (**A**) and premature mortality (**B**) by sex, age group, and clinical health status; Note: Values were HR (95% CI); each model included variables used in the models in Table 3 and Table 4.

In a sensitivity analysis, we investigated the associations of the handgrip strength and weakness on time to mortality by age group and sex. The significant association between handgrip strength levels and the risk of all-cause, and premature mortality was observed in the both age groups and sex (Figure 1). Finally, the unmeasured health status at baseline can affect the relationship between handgrip strength and the risk of mortality, although adjusted for clinical health conditions. Therefore, we also analyzed the association between handgrip strength and an increased risk of mortality after dividing participants who had multimorbidity, and found the results were unchanged (Figure 1).

## 4. Discussion

In this national prospective cohort study, we investigated the association of handgrip strength and weakness on the risk of mortality in Korean adults over 10 years. To our knowledge, this is the first population-based prospective study investigating the association of handgrip strength with the risk of both all-cause and premature mortality in Korean adults. Our findings suggested that muscular weakness, evaluated using handgrip strength, was significantly associated with a higher risk of all-cause mortality in middle-aged and older adults over a 10-year followup period, independent of demographic variables, health-related behaviors, and clinical health conditions. We also found that the handgrip strength was negatively associated with the risk of premature mortality.

Our findings suggested that low handgrip strength was an independent risk factor of accelerated time to mortality with a dose-response relationship. In the present study, the risk of all-cause mortality was approximately two times higher in the lowest handgrip quartile, and 1.56 times higher in the weakness group, based on AWGSOP sarcopenia diagnosis, after adjusting for potential confounding factors. The present findings support and extend the previous epidemiological studies that suggest a relationship between sarcopenia or muscular weakness and the elevated risk of chronic diseases, disability, and mortality [1,20,24,29,30]. However, most of these observational studies considered associations of muscular strength and mortality in older adults or a specific population [1,31,32,33], therefore, limiting conclusions with regard to long-term mortality and premature mortality and muscular weakness in both middle-aged and older adults.

The muscle weakness that accompanies biological aging may also predict chronic disease and decline in physical function [34]. Moreover, higher muscle mass and strength are associated with a reduced risk of cardiovascular disease, frailty, and mortality [3,8,31]. Several previous studies have shown that a lower level of physical function and chronic disease are linked with a higher risk of mortality [35,36]. Critically, muscular weakness might indicate an age-related change of physical function leading to frailty, whilst contributing to increasing the risk of mortality.

Several previous studies have reported the prospective association between muscle strength and mortality [37]. Epidemiological research on English and Brazilian older adults found that muscular weakness as measured by handgrip strength, was linked to a higher risk of mortality [38]. Furthermore, Hamasaki et al. found that in Japanese men with type 2 diabetes, handgrip strength was linked to mortality and hospitalization [18]. In addition, handgrip strength related to an increased risk of mortality, according to longitudinal studies based on 9.5-year follow-up data in the oldest population [39]. Furthermore, a recent review of longitudinal studies investigated the link between loss of muscular mass and/or strength and higher risk of mortality and found that these conditions deteriorated with age [37,40]. Most previous studies have primarily looked at the older population, so information on the contributory relationship between reduced muscle strength and premature mortality in the middle-aged and older population is rare. Our findings demonstrate that muscular weakness is strongly associated with the accelerated time to all-cause and premature mortality, implying that this is an important public health issue that may be improved by including middle-aged persons.

Since it represents a potential loss of life, premature death is an important public health concern [14]. Mortality statistics are commonly used to measure the severity of public health issues and determine the relative relevance of different causes of death. However, since most deaths occur in the elderly, the cause of death is determined by the underlying disease present. Premature death can be used as an alternate measure to reflect the younger age group’s mortality trend [41]. The use of early mortality data as a strategy for determining public health priorities has become more frequent lately [41]. Studies on causes of early death other than underlying disorders, on the other hand, are still limited. Heidi et al., reported that leisure-time physical activity was related to a lower risk of premature death in patients with CKD and type 1 diabetes [16]. Moreover, a U.S. Baby Boomers and Generation Xers study demonstrated that lower cardiorespiratory fitness during middle adulthood was inversely associated with premature death [15]. In addition, in a Swedish national cohort study of 1,547,478 military conscripts, muscle strength, aerobic fitness, and obesity were associated with a higher risk of mortality in adulthood [17]. Taken together, our findings might be used to expand upon the results of previous studies on the association between muscular weakness and premature mortality in the general population.

The key strengths of this study are the utilization of a large and representative sample from the general community. We also considered significant potential confounders. However, there are a few limitations to consider. To begin, we determined muscular weakness based on a handgrip strength, but we did not consider muscle mass, which has previously been described as a key component in predicting clinical health issues. The handgrip test, on the other hand, is a good measure of body function and muscular strength in the middle and older age groups. Lastly, another limitation of this study is that as a result of differences in ethnicity, genetic background, and body size, the results of the present study might not applicable worldwide.

## 5. Conclusions

Our 10-year prospective cohort study showed that muscular weakness measured by handgrip strength was strongly associated with a higher risk of all-cause and premature mortality in middle-aged and older adults. These data suggest that preventing the weakness of muscle strength may contribute to the reduced risk of mortality and premature death.

## Figures and Tables

**Table 1:** Participant characteristics.

	Overall (n = 9229)		Male (n = 4131)		Female (n = 5098)		*p*-Value
Age [years] *^a^*	60.71	±0.11	60.64	±0.16	60.77	±0.15	0.5365
BMI [kg/m^2^]	23.32	±0.03	23.28	±0.04	23.35	±0.05	0.2585
Handgrip strength [kg]	25.53	±0.09	32.52	±0.11	19.86	±0.07	<0.0001
Relative handgrip strength [%]	41.64	±0.12	49.63	±0.16	35.16	±0.13	<0.0001
Muscle weakness [n (%)]	2235	(24.22)	651	(15.76)	1584	(31.07)	<0.0001
Education [n (%)] *^b^*							
<High School	5538	(60.0)	1902	(46.0)	3636	(71.3)	<0.0001
High School	2594	(28.1)	1419	(34.4)	1175	(23.1)	
>High School	1097	(11.9)	810	(19.6)	287	(5.6)	
Smoking status [n (%)]							
Never	6519	(70.6)	1606	(38.9)	4913	(96.4)	<0.0001
Former	877	(9.5)	851	(20.6)	26	(0.5)	
Current	1833	(19.9)	1674	(40.5)	159	(3.1)	
Alcohol consumption [n (%)]							
Never	5569	(60.3)	1457	(35.3)	4112	(80.7)	<0.0001
Once a week	2269	(24.6)	1431	(34.6)	838	(16.4)	
2–3 times/week	835	(9.1)	731	(17.7)	104	(2.0)	
≥4 times/week	556	(6.0)	512	(12.4)	44	(0.9)	
MVPA [n (%)]							
High (≥150 min/week)	2675	(29.0)	1315	(31.8)	1360	(26.7)	<0.0001
Low (<150 min/week)	1037	(11.2)	498	(12.1)	539	(10.6)	
Sedentary (None)	5517	(59.8)	2318	(56.1)	3199	(62.8)	
Clinical health condition							
Obesity	2155	(23.4)	912	(22.1)	1243	(24.4)	0.0092
Hypertension	2469	(26.8)	1004	(24.3)	1465	(28.7)	<0.0001
Diabetes	1075	(11.7)	503	(12.2)	572	(11.2)	0.1545
CVD	420	(4.6)	177	(4.3)	243	(4.8)	0.2694
Stroke	206	(2.2)	118	(2.9)	88	(1.7)	0.0003
Cancer	201	(2.2)	83	(2.0)	118	(2.3)	0.3175

Note: *^a^* Mean ± standard error (all such values), *^b^* Percentage (all such values); *p* values were calculated using *t*-test for continuous variables and chi-square test for categorical variables; MVPA, moderate-to-vigorous physical activity; CVD, cardiovascular diseases.

**Table 2:** Participant characteristics by handgrip strength level.

	Handgrip Strength Level								*p*-Value
	Q1 (n = 2274)		Q2 (n = 2234)		Q3 (n = 2320)		Q4 (n = 2401)		
Age [years]	69.20	±0.19	62.57	±0.19	57.84	±0.19	53.72	±0.18	<0.0001
BMI [kg/m^2^]	22.61	±0.07	23.19	±0.07	23.52	±0.07	23.91	±0.07	<0.0001
Percentage of men [n (%)] b	1009	(44.4)	1064	(47.6)	1026	(44.2)	1032	(43.0)	0.0122
Education [n (%)]									
<High School	1814	(79.8)	1462	(65.4)	1247	(53.8)	1015	(42.3)	<0.0001
High School	334	(14.7)	545	(24.4)	728	(31.4)	987	(41.1)	
>High School	126	(5.5)	227	(10.2)	345	(14.9)	399	(16.6)	
Smoking status [n (%)]									
Never	1593	(70.1)	1532	(68.6)	1658	(71.5)	1736	(72.3)	0.0002
Former	256	(11.3)	240	(10.7)	193	(8.3)	188	(7.8)	
Current	425	(18.7)	462	(20.7)	469	(20.2)	477	(19.9)	
Alcohol consumption [n (%)]									
Never	1582	(69.6)	1350	(60.4)	1334	(57.5)	1303	(54.3)	<0.0001
Once a week	412	(18.1)	549	(24.6)	614	(26.5)	694	(28.9)	
2–3 times/week	134	(5.9)	199	(8.9)	237	(10.2)	265	(11.0)	
≥4 times/week	146	(6.4)	136	(6.1)	135	(5.8)	139	(5.8)	
MVPA [n (%)]									
High	480	(21.1)	652	(29.2)	744	(32.1)	799	(33.3)	<0.0001
Low	202	(8.9)	237	(10.6)	271	(11.7)	327	(13.6)	
Sedentary	1592	(70.0)	1345	(60.2)	1305	(56.3)	1275	(53.1)	
Clinical health condition									
Obesity	414	(18.2)	480	(21.5)	567	(24.4)	694	(28.9)	<0.0001
Hypertension	835	(36.7)	653	(29.2)	556	(24.0)	425	(17.7)	<0.0001
Diabetes	406	(17.9)	295	(13.2)	219	(9.4)	155	(6.5)	<0.0001
CVD	182	(8.0)	118	(5.3)	68	(2.9)	52	(2.2)	<0.0001
Stroke	97	(4.3)	56	(2.5)	36	(1.6)	17	(0.7)	<0.0001
Cancer	72	(3.2)	49	(2.2)	46	(2.0)	34	(1.4)	0.0006

Note: *p* for difference was calculated using chi-square test for categorical variables; MVPA, moderate-to-vigorous physical activity; CVD, cardiovascular diseases.

**Table 3:** Results for the associations of the handgrip strength and weakness group on time to all-cause mortality.

	Number of People		Number of Deaths		Followup Years		Mortality Rate	Hazard Ratio	(95% CI)
	N	(%)	N	(%)	Mean	(95% CI)	(Per 1000 Person-Years)		
Handgrip strength quartile									
Q1	2274	(24.6)	688	(54.2)	9.1	(9.03–9.25)	33.1	2.06	(1.62–2.63)
Q2	2234	(24.2)	315	(24.8)	10.0	(9.89–10.04)	14.1	1.45	(1.14–1.86)
Q3	2320	(25.1)	166	(13.1)	10.3	(10.26–10.36)	6.9	1.18	(0.91–1.53)
Q4	2401	(26.0)	100	(7.9)	10.4	(10.37–10.45)	4.0	1	(Reference)
Relative handgrip strength									
Q1	2304	(25.0)	576	(45.4)	9.4	(9.28–9.48)	26.6	1.42	(1.17–1.73)
Q2	2313	(25.1)	323	(25.5)	10.0	(9.89–10.04)	14.0	1.24	(1.01–1.51)
Q3	2297	(24.9)	211	(16.6)	10.2	(10.14–10.26)	9.0	0.98	(0.79–1.22)
Q4	2315	(25.1)	159	(12.5)	10.3	(10.26–10.36)	6.7	1	(Reference)
Muscle weakness									
Not weak	6994	(75.8)	638	(50.3)	10.2	(10.16–10.23)	7.5	1	(Reference)
Weak	2235	(24.2)	631	(49.7)	9.2	(9.14–9.35)	79.0	1.56	(1.36–1.78)

Note: All models were adjusted for age, sex, education level, alcohol consumption, moderate-to-vigorous physical activity, and clinical health conditions (obesity, hypertension, diabetes, cardiovascular diseases, stroke, and cancer).

**Table 4:** Results for the associations of the handgrip strength and weakness group on time to premature mortality.

	Number of People		Number of Deaths		Followup Years		Mortality Rate	Hazard Ratio	(95% CI)
	N	(%)	N	(%)	Mean	(95% CI)	(Per 1000 Person-Years)		
Handgrip strength quartile									
Q1	2274	(24.6)	386	(44.7)	9.1	(9.03–9.25)	18.6	2.34	(1.80–3.05)
Q2	2234	(24.2)	239	(27.7)	10.0	(9.89–10.04)	10.7	1.78	(1.37–2.31)
Q3	2320	(25.1)	144	(16.7)	10.3	(10.26–10.36)	6.0	1.35	(1.03–1.76)
Q4	2401	(26.0)	95	(11)	10.4	(10.37–10.45)	3.8	1.00	(Reference)
Relative handgrip strength									
Q1	2304	(25.0)	347	(40.2)	9.4	(9.28–9.48)	16.0	1.50	(1.19–1.88)
Q2	2313	(25.1)	219	(25.3)	10.0	(9.89–10.04)	9.5	1.25	(1.00–1.57)
Q3	2297	(24.9)	161	(18.6)	10.2	(10.14–10.26)	6.9	1.05	(0.83–1.33)
Q4	2315	(25.1)	137	(15.9)	10.3	(10.26–10.36)	5.7	1.00	(Reference)
Muscle weakness									
Not weak	6994	(75.8)	507	(58.7)	10.2	(10.16–10.23)	7.1	1.00	(Reference)
Weak	2235	(24.2)	357	(41.3)	9.2	(9.14–9.35)	17.3	1.58	(1.34–1.85)

Note: All models were adjusted for age, sex, education level, alcohol consumption, moderate-to-vigorous physical activity, and clinical health conditions (obesity, hypertension, diabetes, cardiovascular diseases, stroke, and cancer).

## Data Availability

Data used in this study are available in website of the Korean Longitudinal Study of Ageing.
